# Male Circumcision in the General Population of Kisumu, Kenya: Beliefs about Protection, Risk Behaviors, HIV, and STIs

**DOI:** 10.1371/journal.pone.0015552

**Published:** 2010-12-16

**Authors:** Matthew Westercamp, Robert C. Bailey, Elizabeth A. Bukusi, Michele Montandon, Zachary Kwena, Craig R. Cohen

**Affiliations:** 1 Division of Epidemiology and Biostatistics, School of Public Health, University of Illinois at Chicago, Chicago, Illinois, United States of America; 2 Department of Obstetrics, Gynecology, and Reproductive Sciences, University of California San Francisco, San Francisco, California, United States of America; 3 Center for Microbiology Research, Kenya Medical Research Institute, Kisumu, Kenya; Karolinska Institutet, Sweden

## Abstract

Using a population-based survey we examined the behaviors, beliefs, and HIV/HSV-2 serostatus of men and women in the traditionally non-circumcising community of Kisumu, Kenya prior to establishment of voluntary medical male circumcision services. A total of 749 men and 906 women participated. Circumcision status was not associated with HIV/HSV-2 infection nor increased high risk sexual behaviors. In males, preference for being or becoming circumcised was associated with inconsistent condom use and increased lifetime number of sexual partners. Preference for circumcision was increased with understanding that circumcised men are less likely to become infected with HIV.

## Introduction

Between 2002 and 2006, three randomized controlled trials (RCT) of male circumcision to prevent HIV infection in Orange Farm, South Africa; Rakai, Uganda; and Kisumu, Kenya [1], [2], [3] showed a consistent 60% protective effect for circumcised men from heterosexual contact. These findings corresponded with results from numerous observational studies and prompted the endorsement by the World Health Organization (WHO) and Joint United Nations Programme on HIV/AIDS (UNAIDS) of male circumcision (MC) as a welcome addition to HIV prevention strategies [4]. Since that time, initiatives to introduce widespread, safe, voluntary medical male circumcision (VMMC) services have commenced in several sub-Saharan African communities.

The conduction of RCT activities and the release of the positive results likely impacted the perception of MC in associated communities. Each of the trials monitored changing beliefs and behaviors in participants; however, appreciation of changes in the surrounding population is limited. The following short report provides circumcision prevalence estimates alongside attitude, beliefs, and behavior regarding HIV prevention and MC in a community-based sample of men and women residing in Kisumu, Kenya. This population-based survey was conducted in 2006, just prior to widespread availability of trial results through local media outlets. By comparing our results to observational MC acceptability work done prior to trial activities we discuss changes in prevalence and perceptions and act as a baseline for future study as VMMC services are scaled up.

## Methods

This report utilizes a sub-set of questions included in the antiretroviral therapy impact study (ARTIS) conducted in the Municipality of Kisumu between July and October of 2006. Complete details of the sample, study design, and methodology are published elsewhere[5].

In brief, Kisumu is Kenya's third largest city with a population of approximately 400,000 residents[6]. The majority of Kisumu's population belongs to the Luo ethnic group, one of Kenya's largest and the only major group that does not traditionally practice circumcision. The study sample was selected by multi-stage random sampling with cluster definition and systematic sampling followed by random household selection. All men and women aged 15 to 49 years who slept in a selected household the night before the first study visit were eligible for study participation.

The final study protocol, consent and questionnaire were approved by the ethical committees of the Kenya Medical Research Institute and the University of California San Francisco. Written informed consent was obtained for all participants followed by face-to-face interview regarding sociodemographic characteristics, sexual health, and MC/HIV related knowledge attitude and belief (KAB) with questions based on those developed in prior studies in this population[7]. Consenting participants provided venous blood for herpes simplex virus 2 (HSV-2) and HIV testing. Male participants were asked to consent to a visual genital exam to check circumcision status and the presence of genital ulcers. All study procedures were conducted in a private location in or near the home.

Data analysis was performed using SAS (Version 9.1.3, SAS Institute; Cary, NC, USA). Descriptive summaries were based on frequencies and proportions. Associations between variables and HIV seroprevalence are summarized using odds ratios. Non-parametric tests for the differences between groups were used, as appropriate, based on the distribution of continuous variables. To ensure comparability with ARTIS primary study results [5] and the Kisumu arm of the ‘multicentre study’ completed by Buve and collegues in 1998[8], we used a similar overall analytic approach and did not adjust for sampling weights in analyses.

Self reported circumcision status is used throughout, unless specified. All reported odds ratios are adjusted for age, number of sexual partners, ethnic group, and marital status using logistic regression techniques. Any factor showing significant association with a given outcome was assessed for confounding through the comparison of crude and adjusted measures. Any additional factors controlled for are stated in text and tables as appropriate. Analysis of sexual outcomes was limited to participants reporting a history of sexual intercourse.

## Results

Out of 1,050 households visited, 864 (82.3%) had inhabitants eligible for enrollment, resulting in the identification of 2,794 eligible individuals. Of these, 1,833 (65.6%) were contacted and asked to enroll with 178 (9.7%) refusing to participate. The final sample included a total of 1,655 individuals, 749 (45%) men and 906 (55%) women aged 15–49, with 1,508 (91%) giving blood for HIV testing and 1,525 (92%) reporting ever having had sexual intercourse. Just over half (55%) of participants were between the ages 15–24 years and the majority described themselves as Luo (77%) and Christian (95%). Approximately 53% had no more than a primary school education, 36% had some post primary education (e.g. vocational training or secondary school), 6% had attended college or university, and 2% had no formal education.

Seven hundred and fourteen (95%) men provided self reported circumcision status and 262 (37%) consented to confirmatory visual genital exam. By self-report, 535 (75%) men were uncircumcised and 179 (25%) circumcised. By clinical exam, 180 men (69%) were medically defined as uncircumcised, 73 (28%) circumcised, and 9 (3%) were considered ‘abnormally’ or partially circumcised. Twelve men (7%) who self-reported as circumcised were clinically defined as uncircumcised. Two men self-reporting as uncircumcised were clinically defined as circumcised. Considering the classification of ‘abnormally circumcised’ as circumcised, the sensitivity of self-reported circumcision status in this population was 96% and specificity was 99%.

Of participants providing blood for testing, 1,393 (92%) reported having had sexual intercourse. Of these, one hundred and eight males (17%) and 202 females (26%) tested positive for HIV. Two hundred and fifty-two men (41%) and 528 females (68%) tested positive for HSV-2. Three participants reporting no previous sexual intercourse tested HIV positive (1 male and 2 female). All individuals, including these three, reporting no sexual history were excluded from further analysis of sexually transmitted outcomes (i.e. HIV, HSV-2).

### MC status and demographic factors

As expected, circumcision status was strongly related to self-identified ethnic group: 11% of Luo males reported being circumcised compared to 79% of non-Luos (OR  = 32.2, 95% CI 20.0–52.0). Men reporting being circumcised were younger than those not circumcised (mean age  = 25 versus 28 years, p<.001) and more likely to be Muslim (81% [n = 31] of Muslim men reported being circumcised compared to 22% [n = 673] of Christians; p<.0001). The median age at circumcision was 12 years (IQR 8–15 years). Men identifying as Luo reported significantly older age at circumcision (mean age 14) compared to non-Luo men (11 years; p = .005). Table 1 presents demographic factors by preference for circumcision in uncircumcised men.

**Table 1:** Demographic factors and beliefs about HIV by preference for circumcision in uncircumcised men – Kisumu, Kenya.

	Preference to be Circumcisedn = 284 (55%)	Preference to be Uncircumcisedn = 229 (45%)	p-value
**Age**			
Median [range] (SD = )	23 [15–47] (SD = 7.3)	22 [15–49] (SD = 9.2)	0.06
**Ethnicity**			
Luo ethnic group	259 (54%)	224 (46%)	0.001
All other ethnic groups	25 (83%)	5 (17%)	
**Religion**			
Catholic	104 (62%)	63 (38%)	0.02
Muslim	5 (83%)	1 (17%)	
Other Christian	170 (51%)	164 (49%)	
**Region (urban vs. rural)**			
Urban community	120 (59%)	83 (41%)	0.17
Rural community	164 (53%)	146 (47%)	
**Education**			
No formal education	7 (41%)	10 (59%)	0.006
Primary school education	125 (49%)	132 (51%)	
Secondary school education	131 (64%)	74 (36%)	
Post-secondary school	17 (57%)	13 (43%)	
**Alcohol (use in last 4 weeks)**			
Yes	141 (63%)	82 (37%)	0.002
No	143 (49%)	147 (51%)	

### MC preference and belief regarding reduced risk of HIV infection

Over half of uncircumcised men (55%) expressed a preference for being or becoming circumcised and 63% of Luo women expressed a preference for circumcised sexual partners. In the Luo population, the youngest age group (15–19 years) and the oldest age group (40–49 years) were less likely to prefer to be circumcised or to have circumcised partners compared to those aged 20–39 years.

Overall, 55% of participants held the belief that circumcised males are less likely to become HIV infected. This belief was associated with both circumcision status (OR = 2.03; 95% CI 1.2–3.3) and preference in uncircumcised men (OR = 3.15; 95%CI 2.2–4.6). Additionally, Luo women preferring circumcised partners were over six times more likely than other Luo women to believe that circumcised men are less likely to be HIV infected (OR = 6.64; 95%CI 4.5–9.8).

### Association with HIV/HSV-2 seroprevalence, genital ulceration, and sexual risk behaviors

In this study population, circumcision status was not associated with HIV or HSV-2 seroprevalence or current genital ulceration controlling for age, lifetime number of sex partners, marital status, and ethnicity (HIV: OR = 1.0; 95% CI 0.5–2.0; HSV-2: OR = 1.0; 95% CI 0.5–1.7; Genital ulceration: OR = 0.8, 95% CI 0.3–1.8). In uncircumcised men and in women, circumcision preference was not associated with HIV or HSV-2 serostatus. Men favoring future circumcision were, however, two and a half times more likely to report current genital ulceration (OR 2.5, 95% CI 1.3–5.1) than men not favoring circumcision.

By circumcision status, no significant difference between age at sexual debut, lifetime number of sexual partners, or reported condom use was observed. Women preferring circumcised partners did, however, report more lifetime number of partners, (median number 3 vs. 2; Komogorov-Smirnov, p = 0.06). Uncircumcised men with preference to become circumcised were more likely to never or inconsistently use condoms compared to other uncircumcised men (OR = 2.7; 95%CI, 1.6–4.7), and less likely to have an age of sexual debut less than 16 years of age (OR = 0.55; 95% CI 0.4–0.8). Additionally, uncircumcised men who preferred to be circumcised were more likely to report a sexual history including one or more casual partnerships (OR 1.9, 95% CI 1.03–3.6). Table 2 provides further detail for uncircumcised men regarding circumcision preference, associated high risk behavior, and STI infection.

**Table 2:** Association of MC preference in uncircumcised men with STI infection and high risk behaviors.

Factor	Preference to be Circumcisedn = 284 (55%)	Preference to be Uncircumcisedn = 229 (45%)	OR (95% CI)
**HIV Status**			
PositiveNegative	48 (20%)197(80%)	28 (16%)152 (84%)	1.42 (0.8–2.5)†
**HSV status**			
PositiveNegative	108 (44%)137 (56%)	73 (41%)107 (59%)	1.23 (0.8–1.9)†
**Genital Ulcers**			
Current report/visible genital ulcersNo current genital ulcerations	36 (14%)220 (86%)	12 (6%)185 (94%)	2.55 (1.3–5.0)† *
**Condom use**			
Inconsistent useConsistent use	67 (67%)33 (33%)	41 (42%)57 (58%)	2.82 (1.6–5.0) § *
**History of ‘casual’ sex partners**			
History of casual partners	63 (58%)	35 (39%)	1.92 (1.03–3.6)† *
No history of casual partners	45 (42%)	55 (61%)	
**Lifetime number of sex partners**			
Median (IQR)	6 (3–11)	5 (3–9)	P = 0.22‡

*Statistically significant association (p≤.05),

† Controlling for age, number of sex partners, ethnic group (Luo/non-Luo), and marriage status.

§ Controlling for age.

‡ Difference by Kolmogorov-Smirnov test for comparing two groups.

## Discussion

We have briefly described the circumcision prevalence, preference, knowledge, and beliefs in a traditionally non-circumcising community just prior to widespread promotion of VMMC for HIV prevention.

In Kisumu, MC prevalence in Luo men was stable at 10% between 1997 and the period of the current study nearly a decade later[9]. We observed no difference in reported sexual risk-taking behavior between circumcised and uncircumcised men suggesting that no risk compensation was occurring among circumcised men. The majority of men, 75% of those circumcised and 61% of those uncircumcised, understood that circumcised men are less likely to get infected by HIV, and this belief was strongly associated with circumcision preference in men and women.

In uncircumcised men and Luo women there were high levels of circumcision acceptability and preference. Almost 60% of uncircumcised men stated an inclination to be circumcised and over 60% of women reported preferring circumcised partners despite the relatively low potential exposure to circumcised partners in the population. Again, circumcision predilection was associated with the belief that circumcised men are less likely to be infected by HIV, indicating that reduced HIV risk is a potentially motivating factor. This agrees with a 2005 study by Mattson *et al.* finding that circumcision preference in Nyanza would likely be high if the procedure was proven effective[10].

Uncircumcised men who preferred circumcision were more likely to report inconsistent or no condom use, describe sexual partners as ‘casual’, and report current/recent genial ulcerations. This finding reflects conclusions from a 2007 study completed in a similar population by Agot and colleagues that men engaging in riskier sex and reporting more frequent penile injury were more likely to be early adopters of VMMC [11]. Such findings may indicate the self-selection of higher risk individuals for early VMMC adoption potentially leading to greater impact in the community than the biological protective effect of being circumcised [12], [13].

This study did not detect the expected association between male circumcision and HIV seropositivity, possible due to limitations in sample size and prevalence. With 38% of men and nearly 65% of women HSV-2 positive, however, our ability to detect any association between genital herpes and circumcision status or preference for circumcised partners was improved - no association was found. This supports the lack of impact on HSV-2 seroconversion with MC noted in the Kisumu trial [14], but is in contrast to the protective effect noted in the Rakai and Orange Farm trials [15].

Our study had a number of limitations. As with any survey, self report bias can be a significant limitation. Additionally, there is a possibility of misclassification of circumcision status; however, in this population self report had strong agreement with confirmatory clinical exam. Ultimately, our ability to contact just over 65% of eligible participants for enrollment is a limitation and could impact the validity of our findings. Strengths of this study include a randomly selected general population sample, the availability of HIV and HSV-2 test results linked to responses to a structured interview, and a relatively large sample size.

By sampling the population from which the randomized controlled trial of MC in Kenya was conducted, this study offers insight into the early impact of that study on the surrounding community. Considering the encouraging results of that trial, the two similar trials in South Africa and Uganda, the international health community's subsequent endorsement of MC as an HIV prevention measure, and the Kenyan government's national plan for community based VMMC promotion and provision, this work serves as a valuable baseline for subsequent assessments of changes in characteristics, perceptions, uptake, and impact of MC in Kisumu Kenya over the coming years.
